# Supplementary material for: Identification of Freezing-Responsive microRNAs and Their Targets in Chinese Jujube by Small RNA and Degradome Sequencing
Source: Int J Mol Sci. 2026 Sep 4;27(17):7912. doi: 10.3390/ijms27177912 (PMC13566890; doi:10.3390/ijms27177912)
Supplement: Supplementary file 1 [file ijms-27-07912-s001.zip › Supplementary Material S2/novel_miR_164.pdf]

Provisional ID	:	novel_miR_164
Score total	:	25.1
Score for star read(s)	:	3.9
Score for read counts	:	23.1
Score for mfe	:	0.3
Score for randfold	:	-2.2
Score for cons. seed	:	
Total read count	:	57
Mature read count	:	52
Loop read count	:	4
Star read count	:	1

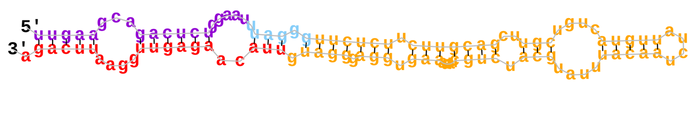

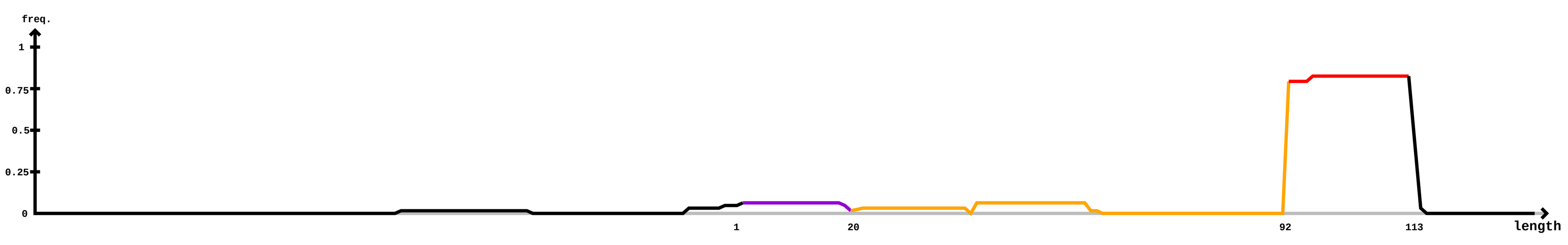

Star

Mature

```
5'- ugaugguaaacaauuauggcauuaguccaaagucucagcacuaugcuugcuugguugauauagugagucgaucaggucagcuagagacagcuugcaauguauccuaacaaugccauuugaagcagacucuggaauuuaggguucucuucuugcagcuugcugucauguuaucuaacauuuaugcaucugcuaaaaaguggagggauguuacaagaguuggaauucagaugaaugaacaagcagucaagaa -3' obs
    ugaugguaaacaauuauggcauuaguccaaagucucagcacuaugcuugcuugguugauauagugagucgaucaggucagcuagagacagcuugcaauguauccuaacaaugccauuugaagcagacucuggaauuuaggguucucuucuugcagcuugcugucauguuaucuaacauuuaugcaucugcuaaaaaguggagggauguuacaagaguuggaauucagaugaaugaacaagcagucaagaa     exp
    (((((.((........)).)))))...............(((.(((.(((((((((((......))))))))))).)))))).(((.(((((((.(((.....))).)).((((((((...((((((......(((.(((((((.(((((((..(((....(((((....)))))....))).))))....))).))).)))).)))..))))))....))))))))......))))).)))..... 
```

read	reads	mm	sample
auagugagucgaucaggucagc	1	0	LT-1
caaugccauuugaagcagacucuggaa	1	0	LT-3
caaugccauuugaagcagacucuggaa	1	0	LT-1
cauuugaagcagacucugga	1	0	LT-3
uugaagcagacucuggaau	1	0	LT-1
uuaggguucucuucuugca	1	0	JCK-3
uuaggguucucuucuugca	1	0	JCK-1
cuuAcugucauguuaucua	3	1	LT-1
cuugcugucauguuaucuaac	1	0	LT-1
uuacaagaguuggaauucaga	22	0	LCK-1
uuacaagaguuggaauucaga	28	0	LCK-2
aagaguuggaauucGgaug	2	1	JCK-2
